# Implicit and Explicit Timing in Oculomotor Control

**DOI:** 10.1371/journal.pone.0093958

**Published:** 2014-04-11

**Authors:** Ilhame Ameqrane, Pierre Pouget, Nicolas Wattiez, Roger Carpenter, Marcus Missal

**Affiliations:** 1 Institute of Neurosciences (IONS), Cognition and System (COSY), Université catholique de Louvain, Brussels, Belgium; 2 Institut du Cerveau et de la Moëlle (ICM), CNRS UMR 7225, Paris, France; 3 Department of Physiology, Development and Neuroscience (PDN), Cambridge University, Cambridge, United Kingdom; Duke University, United States of America

## Abstract

The passage of time can be estimated either explicitly, e.g. before leaving home in the morning, or implicitly, e.g. when catching a flying ball. In the present study, the latency of saccadic eye movements was used to evaluate differences between implicit and explicit timing. Humans were required to make a saccade between a central and a peripheral position on a computer screen. The delay between the extinction of a central target and the appearance of an eccentric target was the independent variable that could take one out of four different values (400, 900, 1400 or 1900 ms). In *target* trials, the delay period lasted for one of the four durations randomly. At the end of the delay, a saccade was initiated by the appearance of an eccentric target. *Cue&target* trials were similar to *target* trials but the duration of the delay was visually cued. In *probe* trials, the duration of the upcoming delay was cued, but there was no eccentric target and subjects had to internally generate a saccade at the estimated end of the delay. In *target* and *cue&target* trials, the mean and variance of latency distributions decreased as delay duration increased. In *cue&target* trials latencies were shorter. In *probe* trials, the variance increased with increasing delay duration and scalar variability was observed. The major differences in saccadic latency distributions were observed between visually-guided (*target* and *cue&target* trials) and internally-generated saccades (*probe* trials). In *target* and *cue&target* trials the timing of the response was implicit. In *probe* trials, the timing of the response was internally-generated and explicitly based on the duration of the visual cue. Scalar timing was observed only during *probe* trials. This study supports the hypothesis that there is no ubiquitous timing system in the brain but independent timing processes active depending on task demands.

## Introduction

The brain devotes a lot of resources to the anticipation of the future state of the world. Consequently, anticipatory movements are often observed before the occurrence of a predictable stimulus. This requires an estimate of the expected time of stimulus appearance and a perception of elapsed time. However, time ‘perception’ is not a single entity but depends on contextual factors like temporal scale, activity or rest, attention [1], [2], emotions [1], [3], [4], pathologies like Parkinson's disease [5], and many other factors (reviews in [6] and [7]). One major difference amongst timing processes that has been established is between their *explicit* or *implicit* nature ([8], [9], [10]). *Explicit* timing refers to the capacity to make an overt intentional decision on the basis of temporal information (e.g.: ‘We should leave in approximately 5 minutes’). In the taxonomy proposed by Coull and Nobre, [11], there is explicit timing if an overt estimation of duration is required. *Implicit* timing refers to the capacity to time actions precisely based on regularities extracted from the environment. For instance, in order to pursue a moving target with the eyes, primates implicitly use temporal information extracted from previous stimulus motion to initiate a smooth eye movement [12], [13], [14].

One particular case of implicit timing is referred to as *temporal expectation* that could build up from an implicit estimate of the changing probability of occurrence of an event [15]. This conditional probability of event occurrence is referred to as the *hazard rate* [16], [17]. In timing studies a button press response is often required to reproduce a particular duration or to indicate when an expected event is likely to occur. The reaction time (RT) of the key press is the dependent variable whose statistics is affected by temporal preparation and decision-making. A typical experiment comprises a warning cue followed after a foreperiod by a stimulus that the subject has to respond to (imperative stimulus). The delay between the warning cue (ready signal) and the imperative stimulus is referred to as the *foreperiod*. The hazard rate hypothesis suggests that expectancy builds up with the changing probability of target appearance during the foreperiod [18]. Some results of foreperiod studies in the oculomotor domain support the hazard rate hypothesis [19], [20], [21], [13]. The hazard rate hypothesis will be referred to as the ‘classical view’. In contrast, an alternative hypothesis suggests that the foreperiod effect on RT is mainly due to the influence of the previous foreperiod on temporal preparation [22], [23]. Expectation concerning the duration of an upcoming foreperiod builds up from the memory of the duration of the foreperiod experienced just before. This hypothesis is usually referred to as the ‘trace conditioning hypothesis’, [24].

Explicit and implicit timing activates different distributed brain networks. Indeed, [24] showed that the anatomical network involved in estimating the duration of an event and predicting when a future event could occur are largely different. Indeed, explicit duration estimation involves a right-sided fronto-striatal network whereas implicit temporal expectation involved mostly the left inferior parietal cortex. However, implicit and explicit timing must act in synergy depending of the information present in the environment. Moreover, different temporal processes could be involved in milliseconds and seconds ranges of durations and be differentially affected in neurological diseases, [25]. Therefore, there is a need for a single experimental approach where implicit and explicit timing processes could be tested using the same motor response in different timing ranges. The saccadic system is particularly appropriate for this approach. Indeed, saccadic latency is extremely sensitive to the nature of the stimulus in the temporal and spatial domains. However, the influence of the explicit or implicit temporal nature of a task on saccadic latency distributions is still poorly understood. If saccadic latency distributions have different characteristics in explicit and implicit oculomotor timing tasks, this would be strong behavioral evidence in favor of the multiplicity of timing systems in the brain.

In the present study, we developed a simple paradigm where the same movement, a saccade, could be implicitly timed by the appearance of a visual target or be explicitly initiated on the basis of a visual cue and internally-generated. The use of saccadic latency as dependent variable allows a direct comparison of explicit and implicit timing processes in the same range of durations with the same motor response.

## Methods

The experiment was conducted in accordance with the local Ethics committee and approved by the CERES, « Conseil d'évaluation éthique pour les recherches en santé » of the University Paris Descartes, France (IRB number 20122800001072). Participants provided their written consent to participate in this study. This consent procedure was approved by the Ethics Committee. Data is available to participants upon written request.

### Subjects

Ten healthy subjects, 6 females and 4 males, participated in the study (mean age: 31 yrs ; SD: 6.5; range : 19–45 yrs). The results of one subject were removed because he showed abnormally long visually guided saccades latencies (>400 ms on average). All participants were informed about the purpose of the study and procedures before they gave their consent. They all had normal or corrected to normal vision and were neurologically healthy.

### Apparatus

Subjects sat in darkness, facing a CRT screen, which presented stimuli at a frequency of 60 Hz. An EyeLink 1000 infrared eye tracking system (SR Research, Mississauga, Ontario) was used to record movements of the right eye at 1 KHz. All experiments were run with a homemade stimulus generation software based on a real time linux kernel (Xenomaï). Stimulus display and oculomotor data collection were synchronized on a frame-by-frame basis. Saccades were detected offline in MATLAB (MathWorks, Natic, MA) with a velocity threshold of 30 deg/sec.

### Target trials

**Figure 1:**
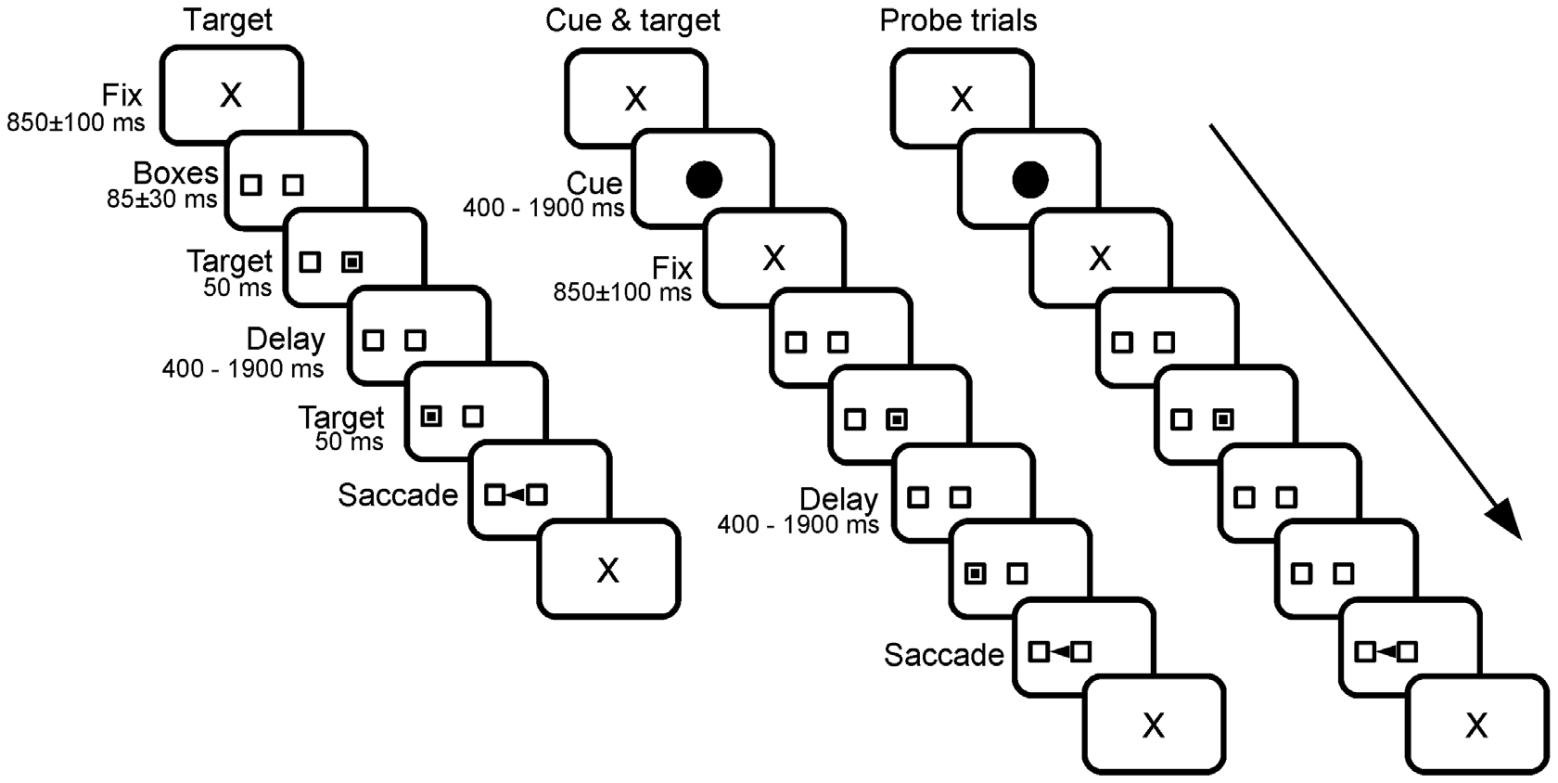
Schematic drawing of the sequence of events in the different trial types presented to subjects. *Target trial*: The trial started with the appearance of a fixation cross for a randomized duration followed by the appearance of two empty ‘boxes’, one at the center of the screen and at a 9-deg eccentric position. After the appearance of the two boxes, a target was flashed in the central one for 50 milliseconds. Extinction of the central target marked the beginning of the delay period that could last either 400, 900, 1400 or 1900 ms. At the end of the delay period, a target appeared for 50 ms in the eccentric box and the subject had to make a saccade to the eccentric box within a 400 ms grace period. *Cue&target trial*: The trial started with a fixation cross (same duration as *target* condition) that was followed by the *cue* period when a red disk was presented on the screen for one of the four durations tested randomly. A short fixation period followed disk appearance and the two empty boxes appeared on the screen. The end of the trial was similar as in a *target* trial but *delay* duration was always equal to *cue* duration. *Probe trial*: same as sequence as *cue&target* trial but the eccentric target appeared with a fixed probability at the end of the delay period.

The trial started with an initial fixation period (referred to as ‘initial fixation’), with a small cross (0.7 deg) appearing on the CRT screen for a random duration (850±100 ms; see ‘X’ on Fig. 1). At the end of this random delay, two empty square ‘boxes’ appeared on the screen (1.4×1.4 deg), one in the center of the screen and one 9 deg eccentric, randomly to the right or to the left. Given that experiments were designed to study temporal processing during the delay period without interference of spatial processing (e.g. spatial working memory), the empty boxes were displayed to provide upcoming target position beforehand.

Afterwards, a square target (1.4×1.4 deg) was flashed in the central box for 50 ms. Extinction of the initial target indicated to subjects the beginning of the *delay* period. Subjects were required to hold on fixation of the central box until another target (1.4×1.4 deg) was briefly presented for 50 ms in the eccentric box. In the variable foreperiod design, the delay period could take one of 4 different values with the same probability: 400 ms, 900 ms, 1400 ms and 1900 ms. In the fixed foreperiod design, the delay period could take only one value determined randomly at the beginning of each block of trials. Subjects were simply required to wait until stimulus appearance in the eccentric box to make a visually-guided saccade.

### Cue&target trials

In *cue*&*target trials*, a visual cue (red disk, 2 deg diameter) was presented at the center of the screen during the initial fixation period for a period lasting either 400 ms, 900 ms, 1400 ms, 1900 ms randomly with the same probability. At the end of cue presentation, the central fixation cross reappeared for a short random delay period before the appearance of the same empty ‘boxes’ as used in *target* trials. A target was flashed in the central box for 50 ms. Extinction of the initial target indicated the beginning of the *delay* period. The delay period lasted for the same duration as the visual cue previously presented. Subjects were required to hold on fixation of the central box until the peripheral target was briefly presented for 50 ms in the eccentric box. As in target trials, saccadic latency was measured with respect to the appearance of the eccentric target. Subjects were required to wait until stimulus appearance in the eccentric box to make a visually guided saccade.

### Probe trials

*Probe* trials were similar to *cue*&*target* trials, except that the eccentric target never appeared at the end of the delay period. Subjects had to use the duration information stored in memory (cue duration) to decide when to overtly initiate a saccade. Subjects were not informed by that a particular trial was going to be a target trial or a probe trial. Total trial duration was always kept the same independently of cue duration. Indeed, saccades could be prompter with shorter cue durations because of the expected closer proximity of trial end for short delays compared to longer ones. Trial end could be considered as a subjective ‘reward’ and longer cues (and delays) could be associated with later rewards and decreased motivation. Therefore, trial length was kept the same for all cue and delay durations by increasing the duration of a final fixation periods during shorter trials so as to counterbalance the increase in trial duration due to cue and delay durations in longer trials.

### Procedure

Data was collected on different days to avoid excessive fatigue of subjects.

- Day 1: three blocks of 100 *target* trials were collected per subject; the four durations used in the present study (400, 900, 1400, 1900 ms) were randomly interleaved in each block of trials (variable foreperiod paradigm).

- Days 2–5: each recording session started with the presentation of a block of 100 *cue*&*target* trials during which subjects were informed that the eccentric target was always going to appear at the end of the delay period; afterwards, 15 blocks of *probe* trials were presented with the probability of target appearance at the end of the delay period taking one out of five values [P(*probe* trial) = 0, 0.25, 0.50, 0.75, 1]. In all blocks of trials, the four durations used were randomly interleaved (400, 900, 1400, 1900 ms). Three blocks of 100 trials were collected for each probability value and each subject. Given the restricted time available for data collection (4 days for 5 probabilities), 2 different probabilities had to be tested on the same day.

- Days 6–7: in six subjects amongst the 10 subjects of the present study, single duration blocks of *target* trials (total of at least 200 trials/duration) were also collected for comparison. A single delay duration was selected for all trials in a block (fixed foreperiod paradigm). Blocks with different durations were presented randomly.

Saccadic latencies longer than 1000 ms in the *target* and *cue*&*target* experiments were not considered for further analysis.

### Statistical analyses

All analyses were performed using the univariate General Linear Model analysis of variance (ANOVA) in SPSS (SPSS, International Business Machines, Armonk USA). The significance threshold α for all analysis was 0.05. Subject identity was used as a random factor to take into account the influence of uncontrolled variability observed between subjects. Fixed factors will be given in the text for each analysis. The dependent variable measured was saccadic latency. In *target* and *cue*&*target* trials, saccadic latency could be defined as the time elapsed between the appearance of the eccentric target (end of the delay) and movement onset. Positive values represent the latency of movements initiated after target appearance. Given that this latency is measured relative to target onset it will be referred to as ‘relative latency’. Saccadic latency could also be measured as the time elapsed between the onset of the delay period and saccade onset. This will be referred to as ‘absolute latency’. In *probe* trials, there was no eccentric target and subjects had to internally generate a saccade after a delay previously indicated by the duration of the cue. Here also, saccadic latency could be computed in two different ways. Indeed, saccadic latency could be measured relative to the end of the delay that the subjects had to time with a saccade (‘relative latency’) or as the time elapsed since the beginning of the delay (‘absolute latency’).

## Results

### Target trials

**Figure 2:**
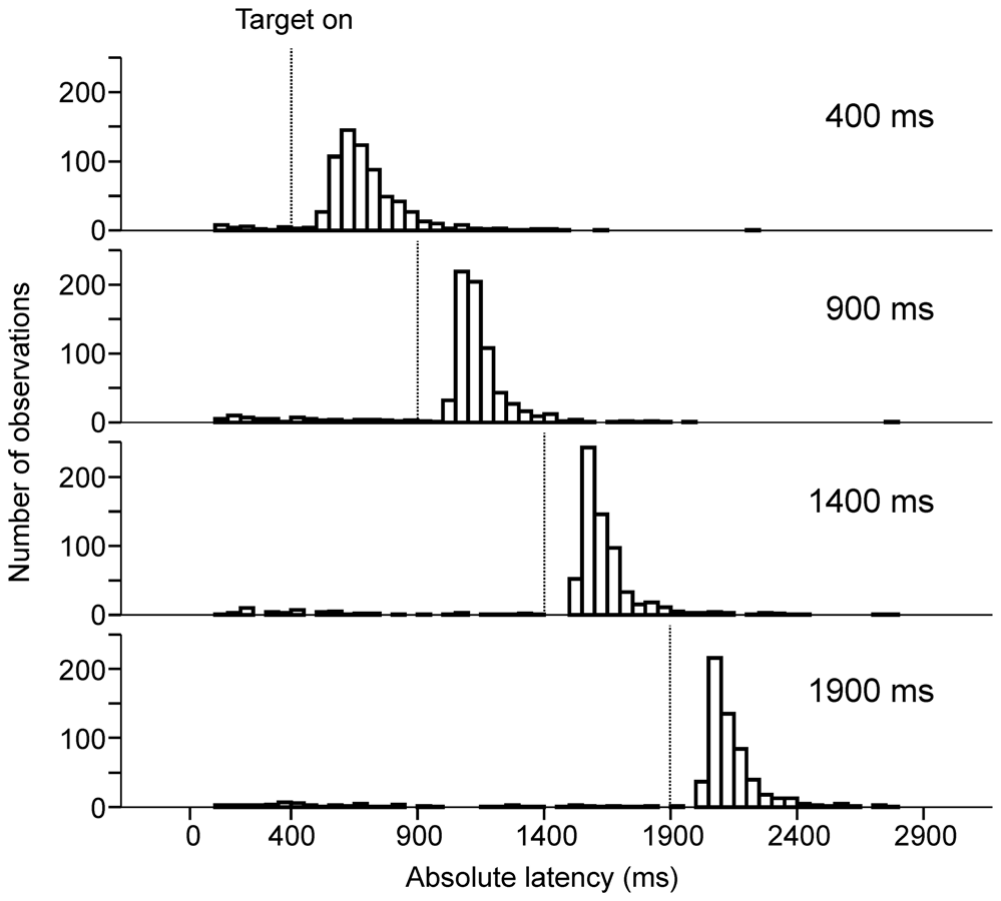
Histograms of saccadic absolute latencies in *target* trials. Time zero on the abscissa represents the beginning of the delay period. The time elapsed until the appearance of the eccentric target is represented with *vertical dashed lines* for the four different durations tested. The ordinate represents the number of saccades in the 10-ms bins.

**Figure 3:**
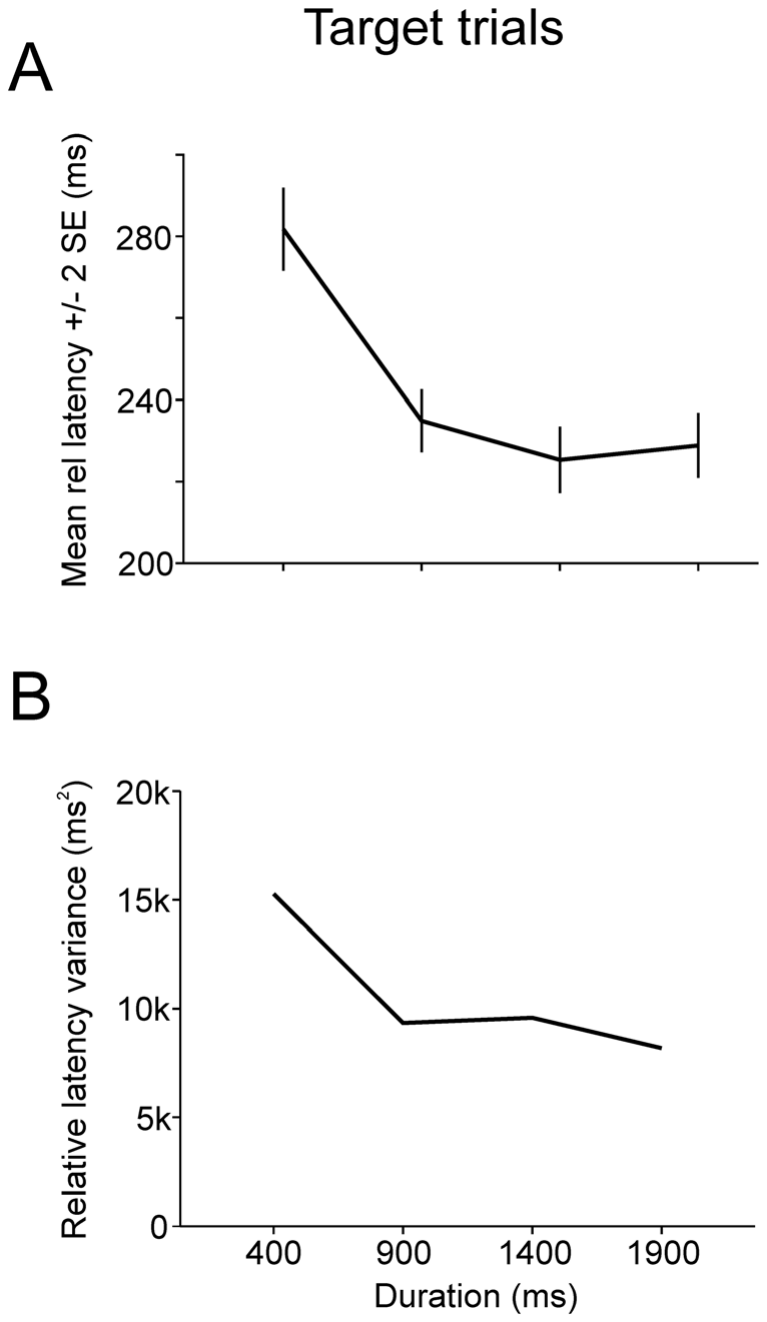
Group data target trials. *A*: Mean relative latency (±2 SE) as a function of delay duration in *target* trials. *B*: Latency variance as a function of delay duration.

During *target trials*, subjects waited for stimulus appearance before initiating a visually guided saccade to the eccentric target. Any influence of delay duration on reaction time should be implicit, given that the task of the subject was simply to make a visually guided saccade without any additional instruction. Figure 2 shows the absolute latency distributions of saccades to the eccentric target (*vertical dashed lines*). Absolute latencies were measured with respect to the offset of the central target. Latency distributions are shown on figure 2 for the four different delay durations tested (400 ms, n = 588; 900 ms, n = 629; 1400 ms, n = 576; 1900 ms, n = 519; group data from all 9 subjects pooled together for the four different delay durations tested). Relative saccade latency was on average 243±106 ms (mean±standard deviation, n = 2312, 9 subjects; median 214 ms). These observations are quantitatively represented on figure 3. Figure 3 shows the influence of elapsed time during the delay period on movement mean relative latency and variance. Mean saccadic latency was higher during 400 ms delay trials and then regularly decreased. Moreover, the variance of latency distributions also regularly decreased with increasing delay duration (Fig. 3 *lower*). These results could suggest that the hazard rate of target appearance altered relative saccadic latency. Indeed, as time elapsed during the delay period, the probability of target appearance changed. Theoretically, the uncertainty about the time of target appearance was larger at the beginning of the trial and then decreased regularly. This decrease of uncertainty was associated with a shorter reaction time, as already shown in various studies since [18]. In the present study, a significant effect of increasing delay duration on latency was also found [One-way ANOVA, delay duration as fixed factor, subjects as random factor; F_3, 24_ = 29.67; p<0.01]. Table 1 shows group statistics for the 4 different delay durations tested. Linear regression analysis revealed that saccadic latency significantly decreased with increasing delay duration in all subjects (p<0.01) except subject GB (p = 0.34).

**Table 1:** Summary of descriptive statistics.

Trial type	Duration (ms)	N	Median relative latency (ms)	Average relative latency (ms)	Average absolute latency (ms)	STD	Variance (ms^2^)	CV
Target (r)	400	588	254	282	682	124	15346	0.18
	900	629	212	235	1135	97	9343	0.09
	1400	576	199	225	1625	98	9600	0.06
	1900	519	202	228	2128	90	8060	0.04
Target (b)	400	1089	164	171	571	78	6059	0.14
	900	1059	175	190	1090	79	6292	0.07
	1400	1093	193	208	1608	78	6114	0.05
	1900	1098	188	202	2102	63	3922	0.03
Cue&targ (r)	400	878	204	218	618	90	8068	0.15
	900	819	191	206	1106	65	4242	0.06
	1400	811	191	205	1605	64	4049	0.04
	1900	800	191	203	2103	64	4042	0.03
**Cue&targ (r)**	400	331	67	84	484	176	31099	0.36
	900	314	64	41	941	213	45560	0.23
	1400	326	−88	−116	1284	330	109066	0.26
	1900	326	−149	−218	1682	531	153817	0.32
**Probe (r)**	400	334	88	132	532	230	52823	0.43
	900	299	91	105	1005	311	96824	0.31
	1400	345	−49	−51	1349	381	145535	0.28
	1900	310	−192	−225	1675	461	212078	0.28

Group data of all subjects. *N*, sample size; *STD*, standard deviation, *CV*, coefficient of variation (STD/mean absolute latency); *(r)*, randomized durations blocks of trials, variable foreperiod; *(b)*, single duration blocks of trials, fixed foreperiod. **Bold type** is used to indicate trials collected in blocks with P(*probe*) = 0.5.

**Figure 4:**
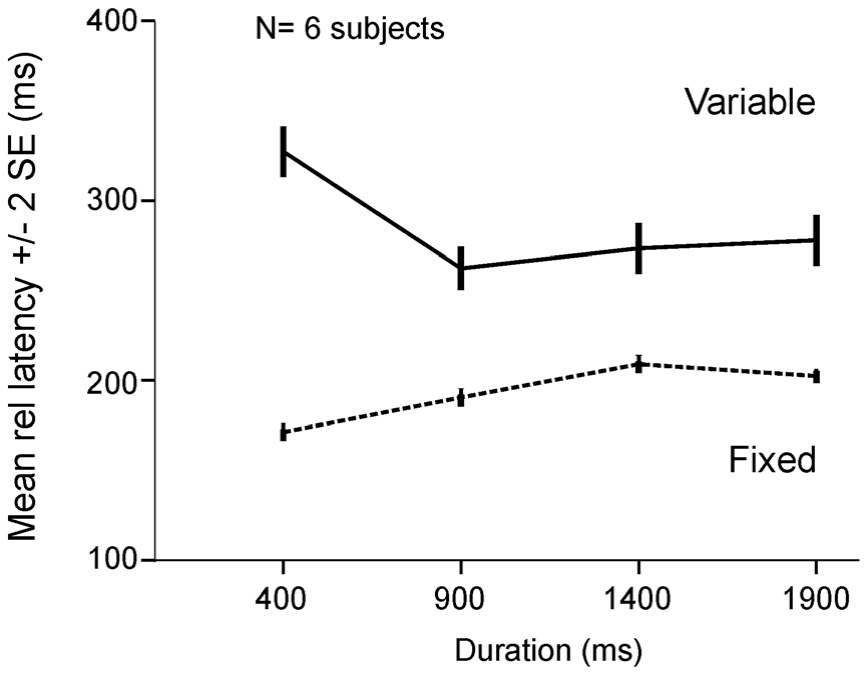
Comparison of variable and fixed foreperiods. Mean latency (±2 SE) as a function of delay duration in *target* trials. Variable foreperiod (*Variable*) and fixed foreperiod blocks of trials (*Fixed*). In the variable foreperiod condition, mean latencies were longer for 400 ms delay duration. An opposite trend was found in the fixed foreperiod condition. Group data from 6 subjects (6/9) who participated in this control experiment.

The decrease of saccadic latency with increasing delay duration was related to the randomization process. Indeed, in a control experiment with 6 subjects, the same four delay durations were tested and compared between the variable and fixed foreperiod paradigms. A two-way ANOVA was applied with delay duration and foreperiod type (fixed or variable) as fixed factors and subject as random factor. A significant main effect of foreperiod type was found [F_1,5_ = 61.4; p<0.01]. On average, latency was shorter in fixed foreperiod trials. The analysis revealed also a significant interaction effect between foreperiod type and duration [F_3,15.019_ = 17.1 ; p<0.01]. Delay duration did not affect latency similarly in fixed or variable foreperiod blocks of trials. Figure 4 shows a summary of this data (group data). On average mean saccadic latencies were shorter with the fixed foreperiod paradigm (*dashed line* on Fig. 4) but, more importantly, mean latency significantly *increased* with delay duration, a trend opposite to what was observed with the variable foreperiod paradigm (continuous line on Fig. 4). These results confirm that the randomization process itself caused the relative latency reduction with increasing delay duration in randomized blocks of trials. Table 1 shows also that the variance of responses was less in blocked trials (fixed foreperiod; *Target (b)* in Table 1) compared with randomized blocks of trials (variable foreperiod; *Target (r)* in Table 1).

### Memory vs hazard rate in target trials

**Figure 5:**
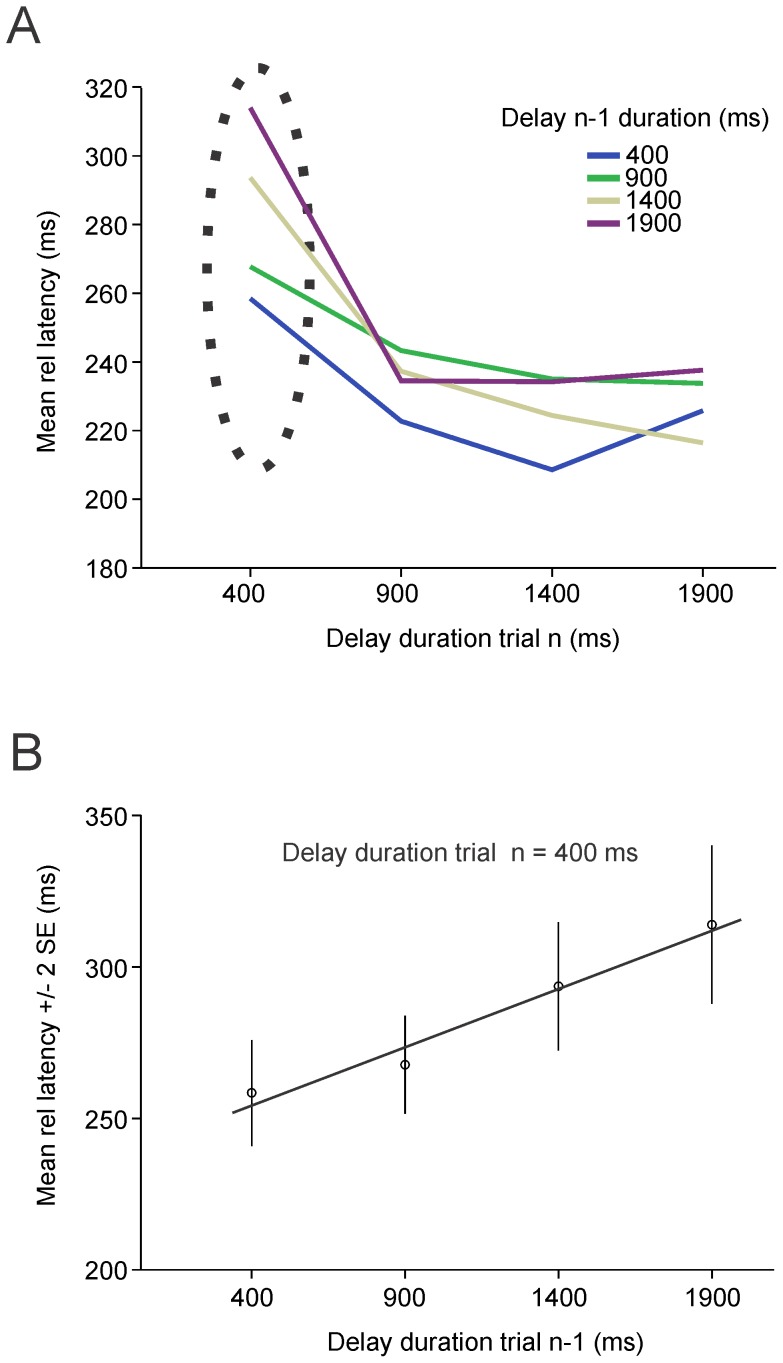
Influence of previous delay duration. *A*: Mean relative latency during the current trial (*‘n’*) as a function of delay duration during the previous trial (*‘n-1’*, *colored* curves). *B*: Data circled with the dashed ellipse in figure *A* (400 ms delay). Group data.

The observed progressive latency reduction with increasing delay duration with the variable foreperiod paradigm could be attributed either to the influence of the changing probability of target appearance as time elapsed during the delay (hazard rate) or to the influence of the memory of the previous delay experienced during the preceding trial. Therefore, the influence of the duration of the previous delay was evaluated. Trials were grouped according to the duration of the previous delay during the preceding trial, trial (*n*-1). For instance, if the duration of the delay during the current trial *n* was 400 ms it could have been preceded by a trial (*n*-1) during which delay duration was similar (400 ms) or longer (900, 1400, 1900 ms). Figure 5 shows the relationship between mean latency (error bars omitted for clarity), delay duration (X-axis) and previous delay duration (colors). These results were analyzed with a two-way mixed ANOVA with current delay duration (trial n) and previous delay duration (trial n-1) as fixed factors and subject as random factor. A significant effect of current delay duration [F_3,24.6_ = 32.4 ; p<0.001] and previous delay duration were found [F_3,25.97_ = 17.1 ; p = 0.001]. Moreover, a significant interaction effect between fixed factors was also found [F_9,83.33_ = 2.4 ; p = 0.016]. Indeed, a post-hoc analysis (Tukey-Kramer post-hoc test of the interaction effect; p<0.001) revealed a significant effect of the duration of the preceding for 400-ms trials only (circled with a dotted oval on Fig. 5*A*). Indeed, for 400 ms delays, saccadic latencies co-varied with the duration of the preceding delay (Fig. 5*B*; linear regression; r^2^ = 0.139; p = 0.001; F[1, 615] = 12.148). This effect was not significant for longer delay durations (more than 400 ms). Therefore, it could be suggested that memory of the previous trial strongly influenced the latency of saccades but for short delay durations only.

### Cue&target trials

**Figure 6:**
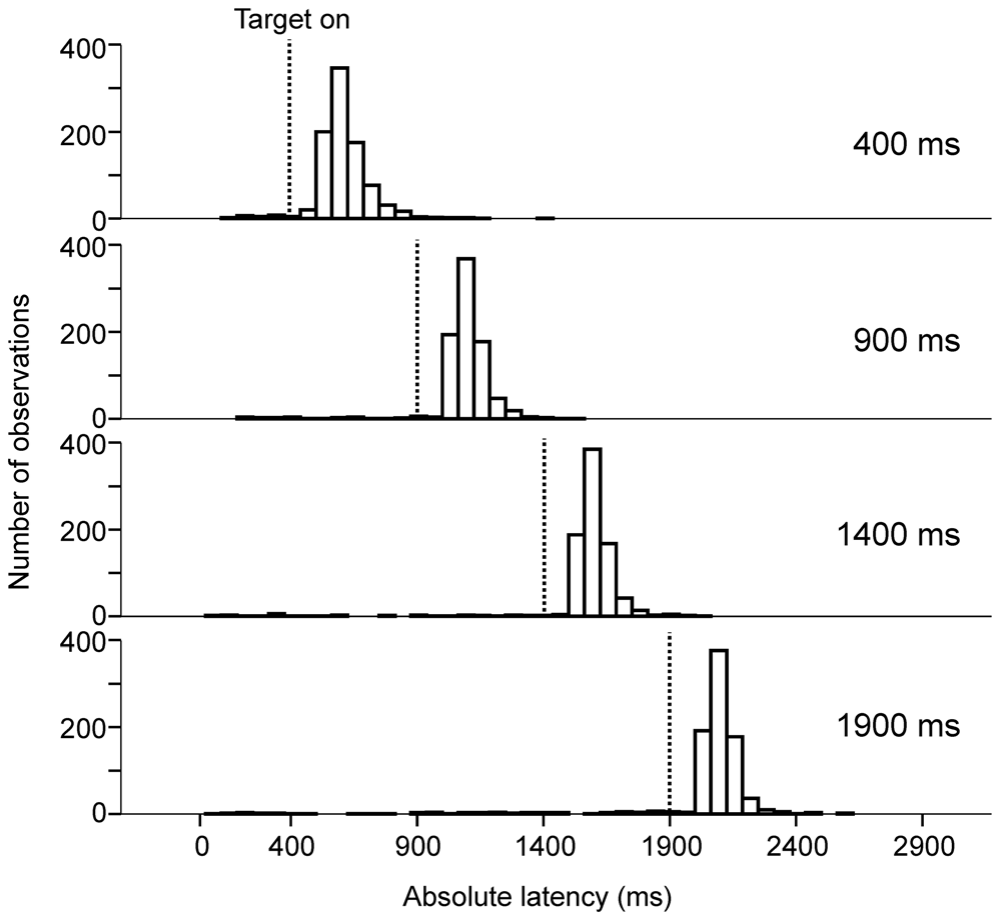
Histograms of absolute saccadic latencies in *cue&target* trials. The ordinate represents the percentage of saccades in the 100-ms bins for each of the 4 delay durations independently. The abscissa represents the time elapsed until the appearance of the eccentric target (*vertical dashed lines*).

**Figure 7:**
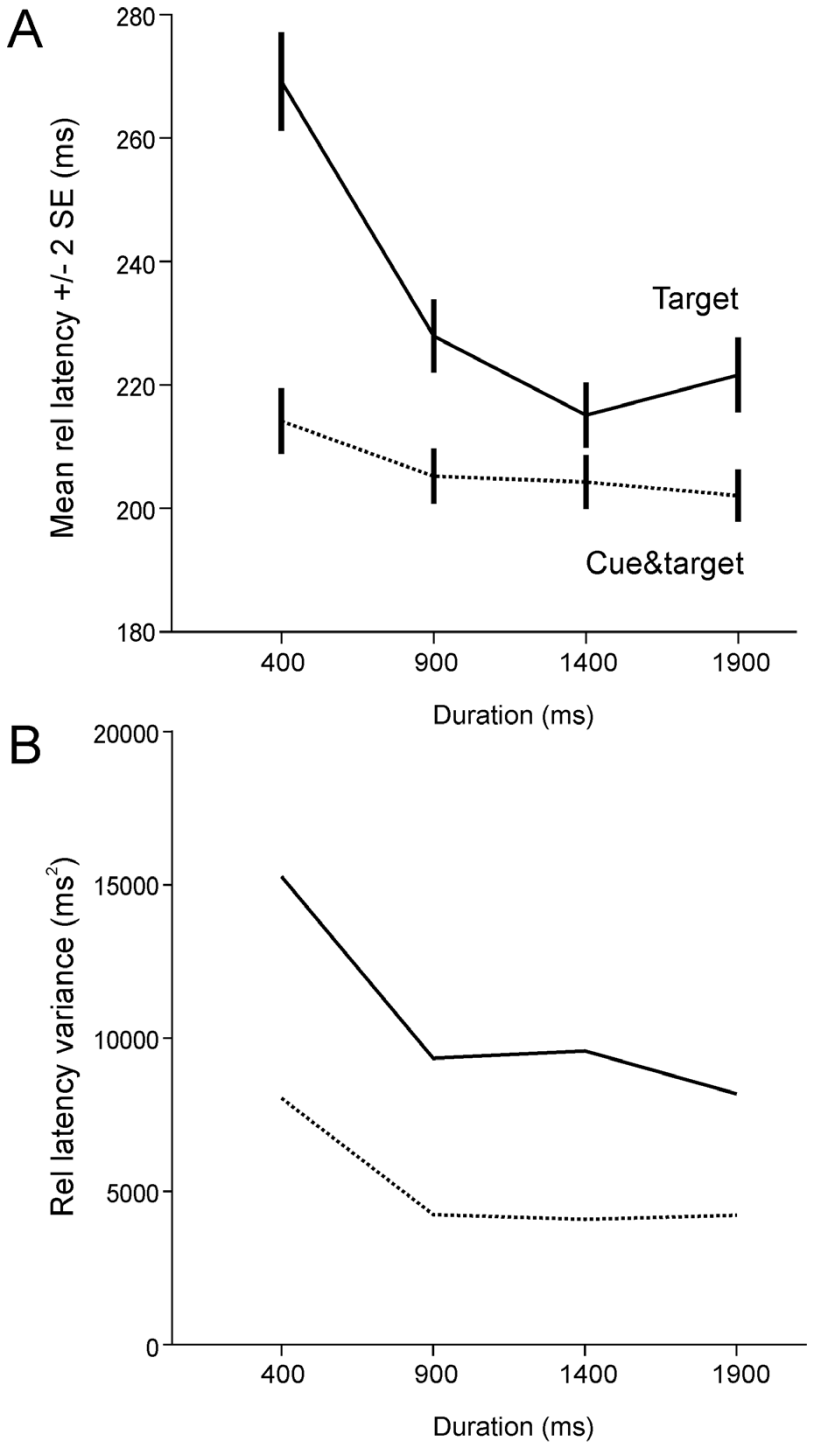
Comparison of *target* and *cue&target* trials. A: Mean relative latency (±2 SE) as a function of delay duration in *cue&target* (dashed line) and, for comparison, *target* trials (continuous line). B: Latency variance in *cue&target* and *target* trials.

In cue&target trials, subjects were informed that a target would always appear at the end of the delay and were informed about the duration of the upcoming delay by the cue. Figure 6 shows the distributions of absolute saccadic latencies (group data). Note that latency distributions appeared very similar to what was observed in *target* trials. Therefore, figure 7 shows the mean relative latency and variance of distributions for both *target* (*solid line*; same data as on fig. 3) and ‘*cue *&* target*’ trials (dashed line). For *cue*&*target* trials, the mean relative saccadic latency modestly decreased with delay duration. However, quantitatively the difference between 400 ms and longer delays was on average quite small (median difference: 13 ms; see Table 1). Results were analyzed with a one-way mixed design ANOVA with delay duration as fixed factor and subject as random factor. For *cue*&*target* trials, the influence of delay duration was not significant [F_3,24.084_ = 2.55 ; p = 0.08].

The mean latency of saccades during ‘*cue *&* target’* trials was on average shorter than during *target* trials. Results were analyzed with a two-way mixed ANOVA with delay duration and trial type (*target* or *cue*&*target*) as fixed factors and subject as random factor. A significant effect of delay duration [F_3,24.064_ = 13.14 ; p<0.001] and trial type [F_1,8.002_ = 13.385 ; p = 0.006] were found. A significant interaction effect between fixed factors was also found [F_3,24.229_ = 21.472 ; p<0.001]. The most interesting influence of the cue was a reduced mean latency and variance in ‘*cue *&* target*’ compared with *target* trials (compare *continuous* and *dashed* curves on Fig. 7). However, in both kinds of trials, the variance was always the largest for the 400 ms duration delay.

In contrast with the *target* trials, no significant effect of the duration of the previous delay was found in *cue*&*target* trials. Results were analyzed with a two-way mixed ANOVA with current delay duration (trial n) and previous delay duration (trial n-1) as fixed factors and subject as random factor. As already shown above, there was no significant effect of current delay duration [F_3,24.108_ = 2.573 ; p = 0.078]. Moreover, no significant influence of previous delay duration was found [F_3,24.437_ = 1.926 ; p = 0.152]. The interaction effect between fixed factors was not significant [F_9,75.257_ = 1.475 ; p = 0.173].

These results suggest that the cue reduced the latency of the upcoming visually-guided saccade. The cue could have evoked an orientation of attentional resources in the time domain.

### Probe trials

**Figure 8:**
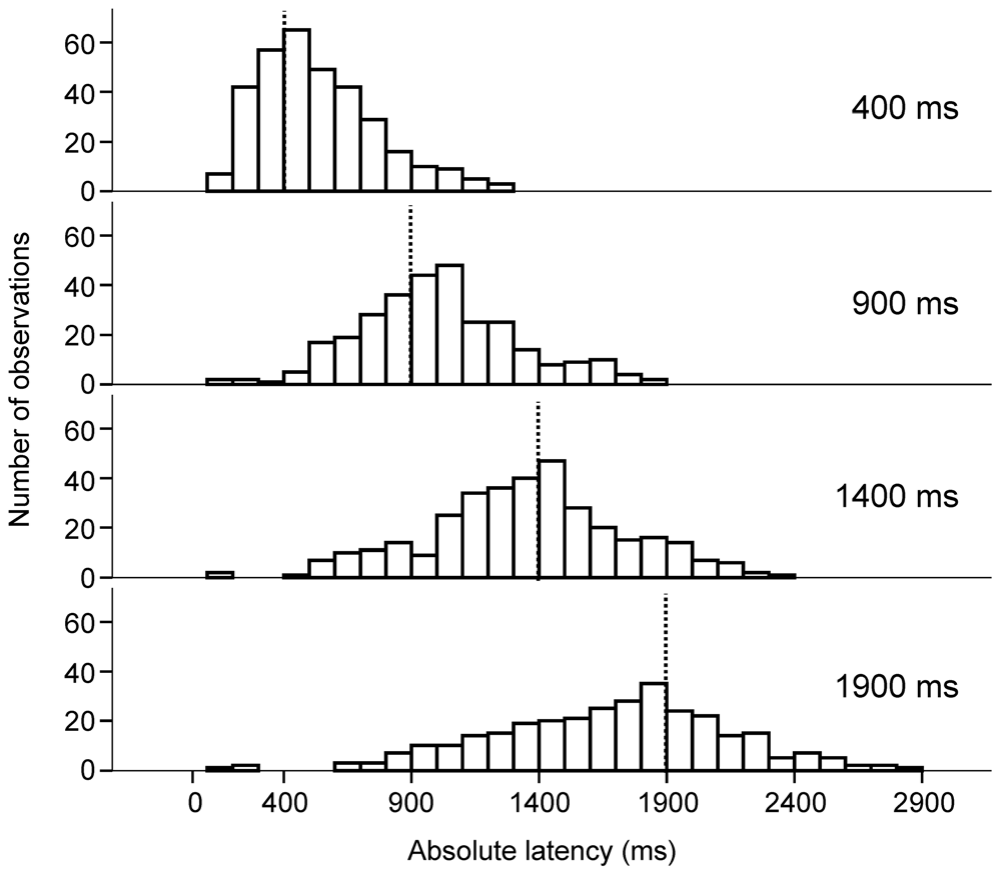
Histograms of saccadic absolute latencies in the *probe* trials. X-axis: saccadic absolute latencies; Y-axis: number of saccades in the 100-ms bins. Note the increasing spread of the latencies with increasing delay duration. Vertical dashed lines: time of target appearance in cue&target trials.

**Figure 9:**
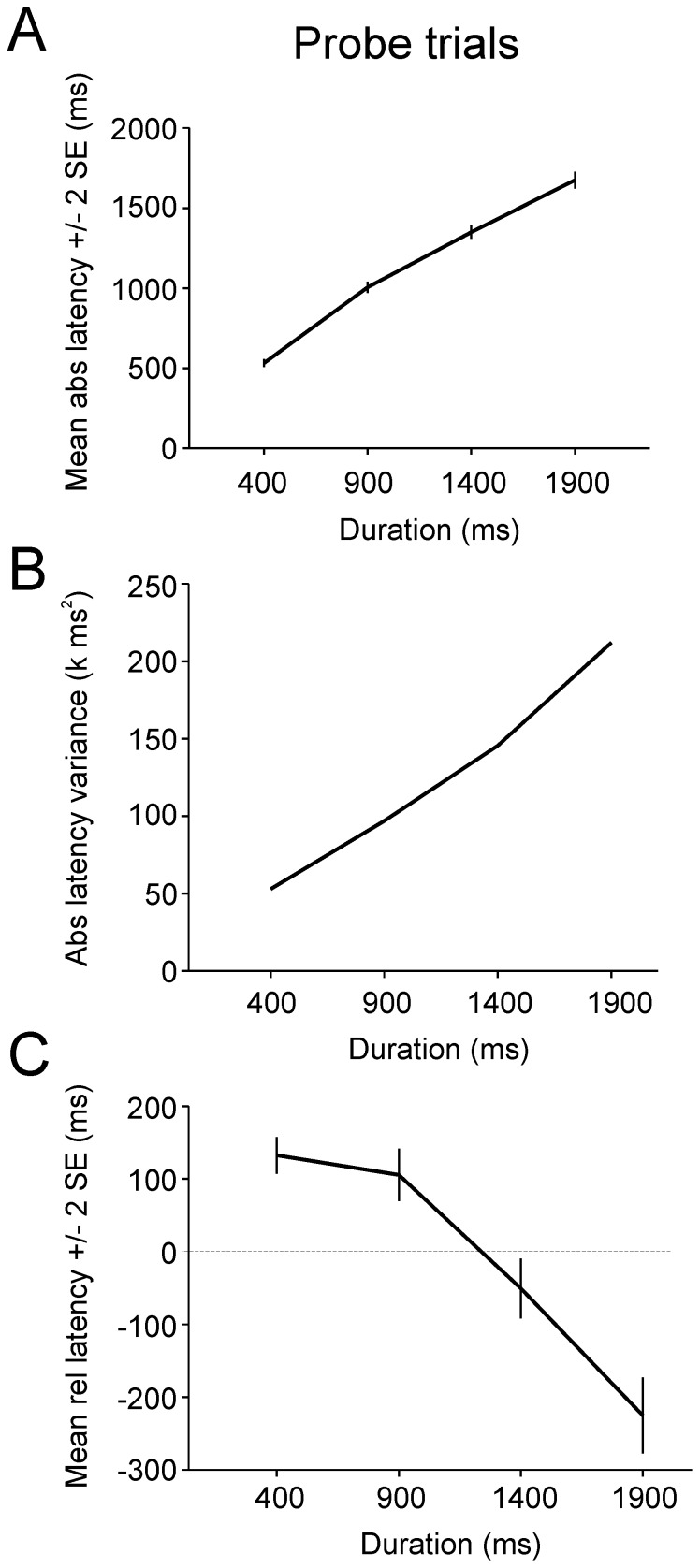
Group data in *probe* trials. *A*: mean absolute latency (±2 SE) as a function of delay duration. *B*: Latency variance for the same data. *C*: Mean relative latency (±2 SE). The horizontal dashed line represents the transition between saccades occurring after the end of the cued duration (positive values) or before (negative values).

During *probe* trials subjects were informed about the duration of the upcoming delay but the eccentric target was never presented. Figure 8 shows the absolute latency distributions during *probe* trials with P (*probe* trial) = 0.5. Vertical dashed lines on the abscissa mark the time of the end of the delay. It can be observed that the peaks of saccadic latency distributions were approximately aligned with the end of the delay period. Moreover, the variance of the distributions increased with cue duration, in clear contrast with what has been observed in the *target* and *cue*&*target* trials. Figure 9A shows the relationship between mean absolute latency and cue (delay) duration. Mean absolute latency increased with delay duration (proportional timing). An analysis of variance with delay duration as fixed factor and subject as random factor showed that saccadic latency was significantly altered by delay duration [F_3, 24 .062_ = 9.262; p = 0.000; see table 1 for samples size]. Figure 9*B* shows that variance of absolute latency increased with the cued durations. There was a significant correlation and a positive linear relationship between latency variance and delay duration (r^2^ = 0.996; F = 470.8; p = 0.002; variance = 47814+61*duration), as predicted by the scalar expectancy theory. These observations illustrate the well-known scalar variability of interval timing and the coefficient of variation of the distributions was approximately constant for delays longer than 400 ms (see Table 1). Figure 9*C* shows mean relative latency as a function of cue duration. For short durations, saccades were initiated after the end of the delay period. This result shows that current delay duration was overestimated (positive values, values above the *horizontal dashed line*). For longer durations, saccades were initiated before the end of the delay period (negative values) and current delay duration was underestimated.

**Figure 10:**
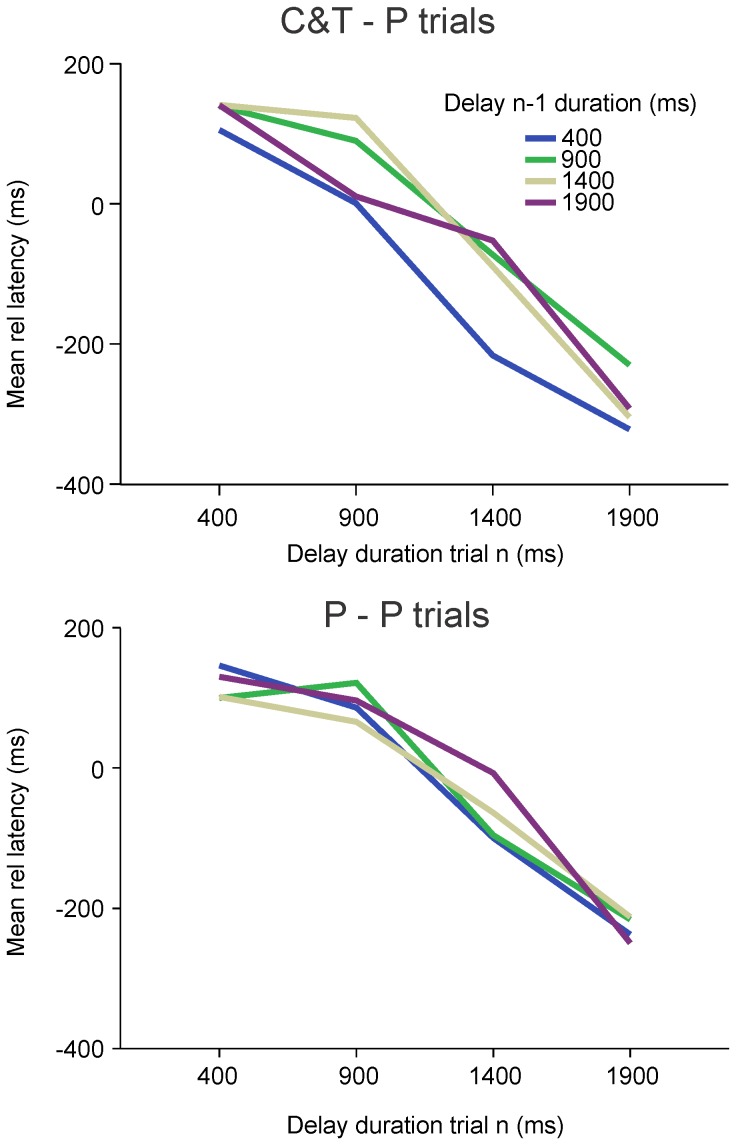
Influence of previous trial type on sequence effect. Y-axis: mean relative latency in milliseconds. X-axis: duration of the delay (cue) during trial ‘n’. Colors: delay (cue) duration during trial ‘n-1’. *A*: *Probe trials* preceded by a *cue&target* trial. *B*: *Probe* trials preceded by a *probe* trial.

In the analysis presented above, probe trial probability was set to 0.5. However, probe probability might be a critical factor affecting characteristics of latency distributions. Therefore, we analyzed results using a mixed model ANOVA with delay duration and *probe* probability as fixed factors and subject as random factor. Duration had a significant effect on saccadic latencies [F_3, 24 .022_ = 11.95; p = 0.000] but *probe* probability did not significantly change saccadic latencies [F_3, 24 .037_ = 2.23; p = 0.111]. Furthermore, no significant interaction between fixed factors was found [F9, 73.169 = 0.331; p = 0.962]. In conclusion, *probe* probability did not change the characteristics of the latency distribution of internally-generated saccades. However, *probe* probability could interact with the hypothetical influence of the previous trial on saccadic latency (sequence effect). Indeed, there could be a sequence effect if a *probe* trial was preceded by a *cue&target* trial but not if was preceded by a *probe* trial. A more vivid memory of previous cue duration would be preserved if the previous trial was a *cue&target* one. Therefore, *probe* trials were classified in 2 groups: the first group contained *probe* trials preceded by a *probe* trial (referred to as ‘P-P’) and the second group contained *probe* trials preceded by a *cue&target* trial (referred to as ‘C&T - P’). If temporal memory was more vivid when a visual target was present during the previous trial, a significant influence of previous trial duration should be found or a significant interaction between current and previous trial duration in the ‘C&T - P’ group (but nothing in the P-P group). An ANOVA was applied on each group separately, with delay duration (trial ‘n’) and preceding delay duration (trial ‘n-1’) as fixed factors and relative latency as dependent variable (subject as random factor). No significant main effect of previous trial duration was found in either the C&T - P [F3, 25.393 = 1.988; p = 0.141] or P-P group [F3, 25.205 = 0.83; p = 0.49]. However, a significant interaction between current and previous trial duration was found in the C&T - P group [F9, 85.613 = 2.132; p = 0.035] but not the P-P group [F9, 75.373 = 1.691; p = 0.106]. These results suggest that there could be a history effect during *probe* trials when the preceding trial was a *cue*&*target trial*. However, a further post-hoc analysis (Tukey HSD) showed that the significant interaction effect between current trial duration and previous trial duration in the C&T - P group was due to a shorter saccadic latency when the preceding trial was a 400 ms *cue*&*target* trial whatever the duration of the current trial. All *probe* trials had a shorter latency if the preceding trial was a 400 ms *cue*&*target* trial (see Figure 10). We interpret this unspecific effect as an arousal influence of a preceding short 400 ms *cue*&*target* trial on the latency of the subsequent saccade.

**Figure 11:**
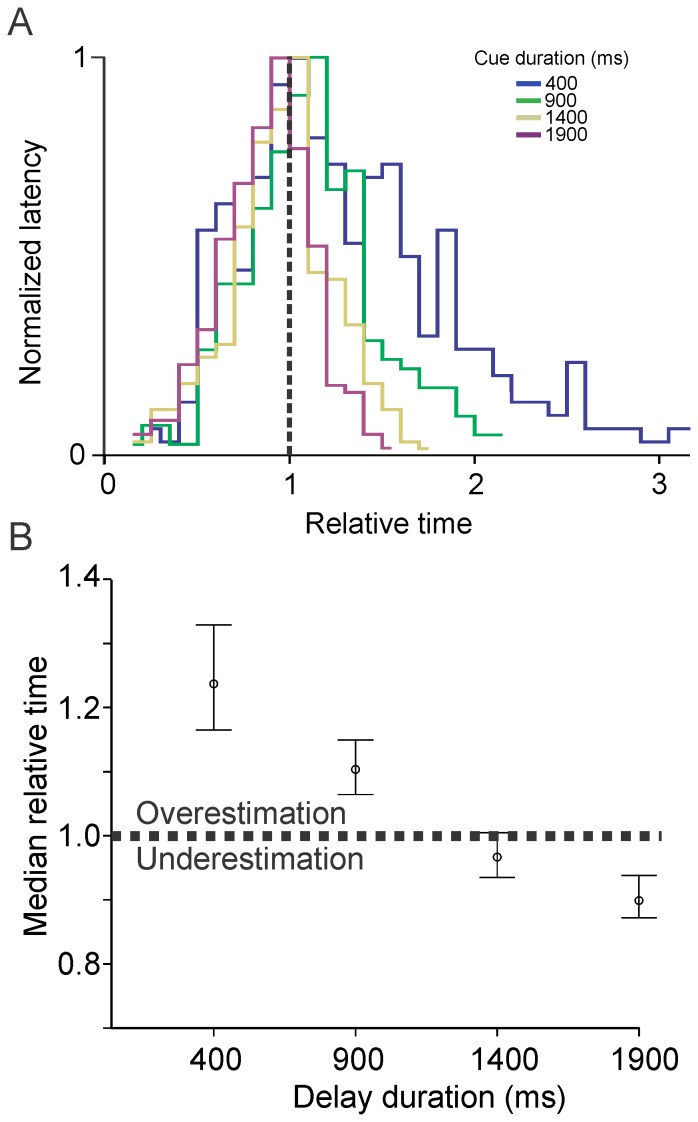
Saccadic latencies in relative time. *A:* Normalized saccadic latencies in relative time (mean saccadic latency divided by delay duration). *B*: Median relative time as a function of delay duration. The horizontal dotted line represents the boundary between overestimation and underestimation.

If plotted in relative time (saccadic absolute latency/delay duration; see Fig. 11*A*) distributions present three important characteristics. Firstly, distributions show a maximum near the time of expected target appearance (vertical dashed line on Fig. 11*A*). The maximum response rate of subjects was close to the time of expected target reappearance indicated by the cue. Secondly, distributions overlap before the end of the delay (rising part of the distributions) but do not overlap thereafter (decaying part of the distributions). Thirdly, short intervals tended to be overestimated and long intervals underestimated (Vierordt's law; see Fig. 11*B*).

### Slope analysis

We applied a variant of the slope analysis developed by D. Getty, [26], to estimate Weber's fraction *k* in timing experiments. Weber's fraction expresses the observation that the variability of subjective time estimations is a constant fraction of the objective interval to be estimated. The slope analysis rests on the assumption that the total variance of responses could be attributed partly to timing processes and partly to other time-independent processes [27], [28]. In this method, *k* is estimated by the slope of the linear relationship between the standard deviation σ of time estimates and target duration *D*, according to the equation:
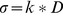
(1)This equation is can be regarded as a generalized form of Weber's law. However, it does not allow for the existence of the time-independent sources of variability mentioned above, whose variance *c* can be taken to add linearly to that due to *D*. Thus:
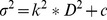
(2)This approach was applied by R. Ivry and R.E. Hazeltine, [28], to a time production task where *D* was the mean intertap interval in a time production task. The *probe* trials in the present study can be described as a saccadic time production task. Therefore, following the same logic, in the present analysis we substituted *D* with the mean latency *L*:
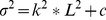
(3)Where the intercept of the linear regression, *c*, represents the variance of time-independent saccadic processes.

**Figure 12:**
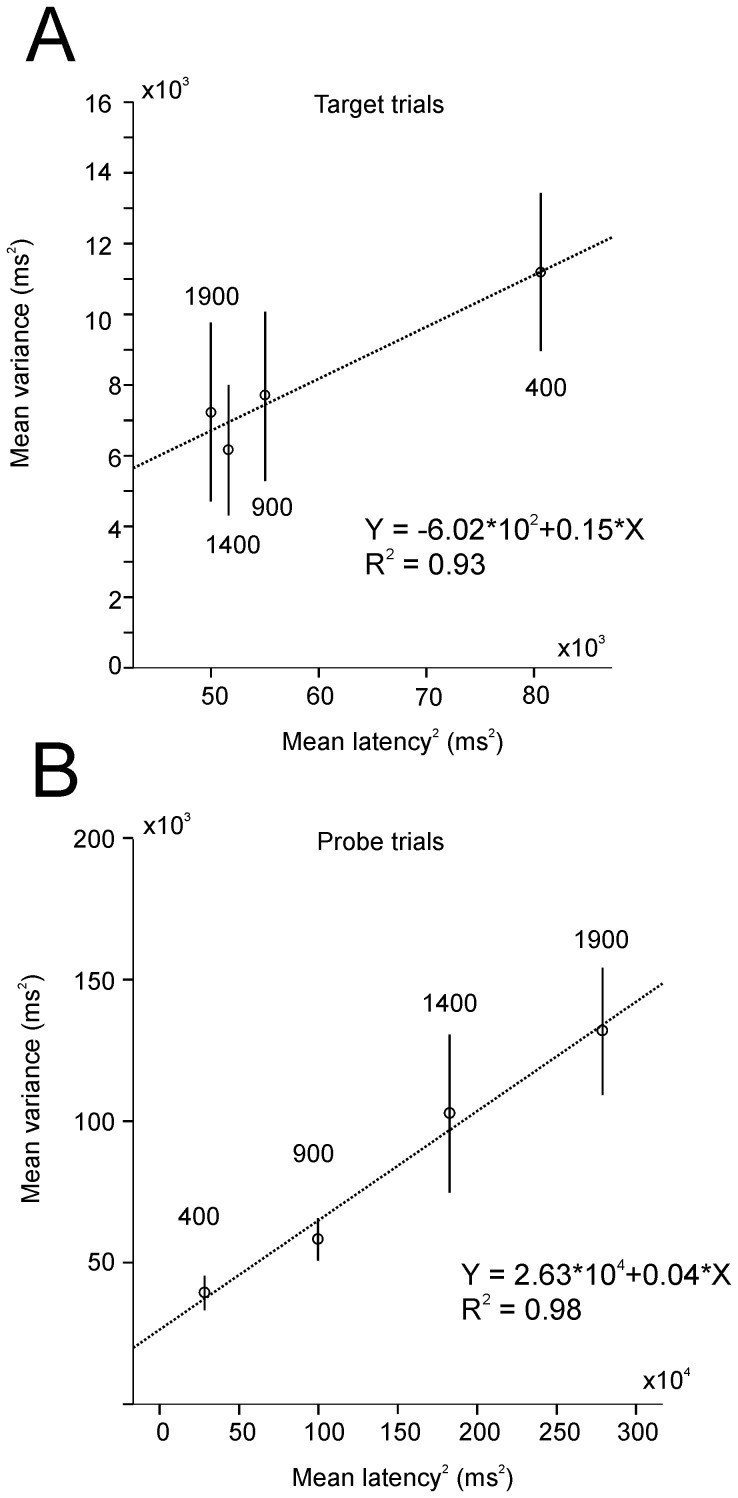
Slope analysis. *A*. Variance as a function of latency squared in *target* trials. *B*. Variance as a function of latency squared in *probe* trials.

Figure 12*A* shows this analysis in *target* trials. There was a linear relationship between *σ^2^* and *L^2^*. However, *σ^2^* and *L^2^ decreased* for increasing delay durations. Therefore, the value of the slope *k^2^* should be interpreted as negative. This result clearly violates the basic assumptions of the slope analysis. Figure 12*B* shows the results for *probe* trials. Mean variance across subjects and sessions is represented as a function of latency squared. The variance of latency increased with the square of latencies according to the equation *Y = *0.04 *L* ^2^+2.63.10^4^. The variance accounted for by the linear fit was high (*R* ^2^ = 0.98). The slope *k^2^* (0.04; *k* = 0.2) was significantly different from zero (regression ANOVA; *P*<0.01). These results reinforces the hypothesis that explicit timing in *probe* trials obeys the general Weber's law but that implicit timing in *target trials* does not.

### Conclusions

The variance of saccadic latency decreased with elapsed time in *target* and *cue&target* trials. In these two types of trials, saccades were visually-guided and timing of the response was implicitly determined by the appearance of the eccentric target. In *probe* trials, subjects had to explicitly time the end of the delay period. These movements were internally-generated on the basis of cue duration and the scalar property was found.

## Discussion

All temporal processing are elaborate and elusive and depend on the context or task in which they are produced. Despite this complexity to be studied, one often suggested hypothesis is the difference between implicit and explicit timing. On the one hand, implicit timing is used to qualify the influence of temporal variables on movement timing although the primary purpose of the task and the instruction given to the subjects is not of a temporal nature per se [29]. On the other hand, explicit timing is used to qualify the fact that the subject is informed about the temporal nature of the task and does voluntarily control the timing of his (her) response. In order to better understand this dichotomy, we used the same movement (a saccade) as response in different contexts, with or without temporal cue and different information provided to subjects. The spatial demand of the task was the same in all experiments, given that the two empty ‘boxes’ indicated the positions of the central and peripheral targets beforehand. In sum, location-specific target expectancies were always the same in all conditions of the present study. This experimental approach was chosen to reduce as much as possible the variability created by other processes not directly related to timing and to allow subjects to focus attention on ‘when’ the target could reappear in the periphery.

In *target* trials with variable foreperiod, we found that movement latency and variance *decreased* as time elapsed during the delay. Moreover, a significant influence of previous delay duration was found, in agreement with the trace conditioning hypothesis. Working memory effects on time perception, within and between modalities, have been observed several times previously, [30], [31]. However, the percentage of the variance explained by previous delay duration on current movement latency was small (r^2^ = 0.139) and limited to 400 ms delays. This result suggests that the trace of the previous delay decays rapidly during the current trial. It has been hypothesized previously that both conditional probability and the preceding foreperiod duration could both influence reaction time with a greater influence of the preceding foreperiod early during movement preparation, [32], [33]. In the present study, adaptation to the hazard rate during the current trial could progressively override the influence of the memory trace of the previous trial. Indeed, at the beginning of the trial, each delay duration is equally likely (P = 0.25). As soon as the short delay had expired without target appearance, the probability of target appearance at the end of one of the other delay periods rose rapidly (P = 0.33). This process of probability updating continues until the probability of target appearance became maximum after 1400 ms when P(delay = 1900 ms) = 1.0.

In *target* trials with fixed foreperiods, saccadic latency modestly but significantly increased with increasing duration of the delay. This is a particular instance of the fixed foreperiod effect [18]. Moreover, we observed also that the variance of latency distributions *decreased*. Therefore, the scalar property of variance [34], [35] was not observed in implicit timing of saccades.

When prior temporal information was provided, two different situations could be distinguished. Firstly, in *cue*&*target* trials, we observed that the variance of latencies was less than in *target* trials and scalar variability was not observed. This reduced variance of responses in *cue*&*target* trials could be due to temporal orienting of attention to the instant of target appearance induced by the cue [1], [36], [2]. This temporal orienting of attention could suppress sequential effects [22], [37]. In the functional taxonomy proposed by J. T. Coull and C. Nobre [11], *cue*&*target* trials could be classified as implicit timing where temporal expectation is deliberately established by pre-cues (endogenous temporal expectation). The influence of the temporal cue was relatively stronger on short durations, as observed previously [38]. Secondly, if subjects were informed that the target could not reappear in a certain proportion of *probe* (‘catch’) trials, saccadic latency distributions had a very different shape and variance always *increased* with increasing delay duration. Scalar variability was observed.

Results of the present study strongly support the hypothesis that the brain uses different timing processes whether prior information is provided and overtly used (explicit timing, *probe* trials) or not (implicit timing, *target* and *cue*&*target* trials; see review in [11]). A previous direct comparison of motor responses of subjects performing an explicit (e.g. finger tapping or intermittent circle drawing) or implicit (continuous circle drawing) timing task found also that the variability of responses were not correlated between tasks, suggesting different neural substrates for explicit and implicit timing [8]. However, Piras and Coull have reported similar Weber fractions in implicit and explicit *perceptual* timing tasks [10]. In the present study, the oculomotor task was as simple as possible. Any influence of elapsed time during the delay on saccade latency must be implicit. Moreover, subjects were not informed that timing of saccades was investigated in *target* trials and were not required either to speed reaction time performance or to respond as quickly as possible. We suggest that the different nature of the tasks (perceptual *versus* oculomotor) could partly explain differences between the Piras and Coull study, [10], and the present one. Moreover, there could exist several implicit timing systems that are actually very different, perhaps even specific for each sensorimotor system, whereas explicit timing would rely on a unified cognitive system, even if it were actually distributed across many brain areas [39], [40].

In the oculomotor domain, Joiner and Shelhamer, [41], used an alternating target paradigm and showed that the timing of predictive saccades was scalar and could depend on an internal clock based on rhythmic behavior. In contrast, the timing of reactive saccades was not scalar. Our results corroborate these findings. In the present study, saccades in the implicit case were reactive, visually-guided saccades and timing was not scalar. Moreover, we have shown that the internal clock for saccades could be based on a flexible representation of the to-be-timed interval in temporal working memory, and does not need to rely on rhythmic behavior. Although rhythmic phenomena are often found in real world situations and could be used to guide prediction, we suggest that the flexibility of saccadic timing requires the ability to use temporal information stored in working memory independently of any repetition of the stimulus.

At a theoretical mechanistic level we suggest that in the implicit case (*target* and *cue*&*target* trials), a simple model with ramp-to-threshold dynamics like LATER (Linear Approach to Threshold with Ergodic Rate, [42]) could explain observed latency distributions. Theoretical models of timing based on a pacemaker could predict responses observed in the explicit case (*probe* trials). However, symmetrical distributions of response rates are expected in relative time [35], [6], [7]. In the present study, the falling edge of latency distributions in relative timing was steeper with increasing delay duration (see Fig. 12). This observation support the hypothesis the threshold for initiating and ending responses could be different and perhaps involve different brain structures also [43].

In conclusion, we used the latency of saccadic eye movements to evaluate differences between implicit (visually-guided responses in *target* and *cue*&*target* trials) and explicit (internally-generated responses in *probe* trials) timing processes. The shape of latency distributions was different in *probe* trials compared with all other conditions and scalar variability was observed. Saccadic latencies are very sensitive to timing and can be used to clearly dissociate its implicit from explicit forms. The present study support the hypothesis that there is no ubiquitous timing system in the brain but independent timing processes and/or brain networks active depending on the context of the task [11], [44], [45] or its automatic *vs* cognitive nature [39]. This study supports the hypothesis that scalar variability is the signature of an overt or explicit estimation of duration but does not hold for implicit timing in oculomotor tasks.
